#  Supporting Nurse Leaders to Recognize and Intervene in Team Members' Suicidality

**DOI:** 10.1111/jnu.70006

**Published:** 2025-03-21

**Authors:** Kristina E. James, Julia Rogers, Rachael Accardi, Gokarna Aryal, Patti Ludwig‐Beymer, Judy E. Davidson

**Affiliations:** ^1^ University of California, San Diego Health La Jolla California USA; ^2^ Purdue University Northwest Hammond Indiana USA

**Keywords:** mental health, nursing leader, role play, self‐efficacy, suicide, workplace wellness

## Abstract

**Introduction:**

Nurses and healthcare support staff have a higher suicide risk than the public. This elevated risk calls for increased efforts to support mental health. Additionally, nursing leaders' education on employee‐specific suicide prevention is lacking.

**Design:**

An evidence‐based project was implemented using the PICO question: Among nurse leaders at an academic healthcare system in California, does the provision of an educational program using role‐playing practice and the creation of a suicide prevention toolkit versus no standard education or training improve self‐efficacy and knowledge on how to take action with a team member who is suspected of being suicidal or voicing suicidal ideation?

**Methods:**

Education sessions were planned based on the literature, with surveys collected preintervention, immediately posteducation, and 1‐month postintervention to assess suicide prevention self‐efficacy and knowledge. Knowledge was measured using a researcher‐constructed questionnaire validated by six suicide prevention experts. The General Self‐Efficacy Scale (range: 10–40) was used.

**Results:**

Sixty participants attended one of 11 scheduled remote‐learning sessions. Mean self‐efficacy significantly improved (pre: 31.3 [*n* = 46, min: 18, max: 40]; immediate post: 33.49 [*n* = 37, min: 24, max: 40]; 1‐month post: 33.77 [*n* = 31, min: 28, max: 40]) (*X* ^2^ = 8.0184, df = 2, *p* = 0.01815). The proportion of incorrect knowledge questions was significantly lower postintervention (mean pre: 24.5%, immediate post: 11.5%, 1‐month post: 10.7*%, X* ^2^ = 23.195, df = 2, *p* = 0.000001). All participants (100%, *n* = 55) recommended the program. Leaders reported feeling better prepared to support suicidal employees.

**Conclusion:**

Project results demonstrate the need to provide suicide prevention training for leaders. The authors recommend requiring training/return demonstration competency as a component of new leaders' onboarding. This program can easily be modified for nurses from prelicensure through senior leadership.

**Clinical Relevance:**

Suicide rates in healthcare members are higher than those of the general population. Suicide prevention programs can help nursing leaders feel better prepared to support and connect at‐risk healthcare workers with resources.

## 1 Introduction

Nurses and healthcare support workers are at higher risk of suicide than the general population (Davidson et al. 2020; Davidson et al. 2024; James et al. 2023; Lee et al. 2022; Olfson et al. 2023). In a retrospective cohort study of US suicides between 2018 and 2021, it was reported that female nurses had up to a 41% higher suicide risk compared to female non‐nurses (IRR 1.21–1.41) (Davidson et al. 2020). Given the known risk of nurse suicide, a call to action has been issued by the Surgeon General, American Academy of Nurses, and National Academy of Medicine and Science (The Surgeon General 2021; Schimmels et al. 2023; National Academy of Medicine 2024). The Centers for Disease Control and Prevention found that healthcare workers are at risk of suicide due to a variety of stressors, including challenging working conditions, emotionally taxing situations with patients and families, job hazards such as workplace violence or risk of exposure to infectious diseases, regular experiences of death and suffering, and availability of lethal means through knowledge and medications (Tiesman et al. 2021). The United States Surgeon General published a report on burnout that highlights the many causes that are plaguing the healthcare workforce and the impacts of burnout, including suicide (The U.S. Surgeon General 2024).

### 1.1 Call to Action

The Joint Commission (2024) National Patient Safety Goal #15 mandates suicide screening for high‐risk patients who arrive with suicidal ideation or a plan. While training may be common in how to identify patients at risk, suicide prevention training specific to the risks of healthcare team members is not standard practice in the healthcare setting. With nurses and healthcare team members being at an increased risk of suicide, nursing leaders are in a perfect position to take action to learn to support team members who are struggling (Davidson et al. 2020; James et al. 2023; Lee et al. 2022; Olfson et al. 2023).

### 1.2 Available Tools

Tools have been created to assist healthcare systems in developing action plans to reduce stigma, provide access, and decrease job stressors that contribute to healthcare worker suicide (American Hospital Association 2023). The American Hospital Association [AHA] (2023) has created a guide for healthcare systems to implement suicide prevention interventions. The American Nurses Association [ANA] (2023) has an informational website that provides resources for nurse suicide prevention and individual resilience skills‐building.

### 1.3 Suicide Prevention Training

Numerous training programs on suicide prevention are available across a variety of sectors, including those targeted to university students and faculty, teachers, pharmacists, pharmacy students, and the public at large (American Hospital Association 2023; Magness et al. 2023; Muehlenkamp and Quinn‐Lee 2023; Sylvara and Mandracchia 2019; Stover et al. 2023; Yonemoto et al. 2019). Program effectiveness has been demonstrated.

A longitudinal mixed‐methods study of a peer‐led suicide prevention program for undergraduates found increases in perceived intervention skills, self‐efficacy, knowledge, and willingness to intervene initially posttraining and at a 3‐month follow‐up (Muehlenkamp and Quinn‐Lee 2023). Qualitative feedback from the participants further affirmed the impact of the program on these findings (Muehlenkamp and Quinn‐Lee 2023).

A study of college and university faculty across the United States surveyed the level of confidence in student suicide prevention (Sylvara and Mandracchia 2019). The study authors found that those faculty who had some training felt more confident in interacting with an at‐risk student than those faculty who did not have formal training in suicide prevention (Sylvara and Mandracchia 2019).

A meta‐analysis of suicide prevention training programs for community and student pharmacists found an increase in the number of suicide prevention programs since 2018, but the impact of the training needs to be measured (Stover et al. 2023). They recommend additional studies be completed to determine the best instructional methods to prepare individuals for engagement in suicide prevention.

Gatekeeper training is a common suicide prevention intervention utilized in the community (Yonemoto et al. 2019). Gatekeeper training is designed to educate clergy members, coaches, teachers, primary and urgent care providers, first responders as well as other community members to identify at‐risk individuals and facilitate connecting them with resources (Yonemoto et al. 2019). A systematic review of gatekeeper training determined that there is a lack of standardization in the training and that the effects of the training are not clear (Yonemoto et al. 2019).

A study of community participants across 21 state‐funded Applied Suicide Intervention Skills Trainings (ASIST) found an increase in participants' suicide prevention self‐efficacy and awareness of risk factors and warning signs, feelings of reduced barriers to helping suicidal individuals, and increased perceptions of controlling the situation (Magness et al. 2023). Study participants filled out the Gatekeeper Training Survey at 6–9 months after training completion to assess self‐reported behavior and utilization of skills learned during the training (Magness et al. 2023). Participants had higher levels of self‐efficacy and decreased perceptions of barriers to responding to a suicidal crisis after attending the ASIST training (Magness et al. 2023).

### 1.4 Organized Nurse Leader Onboarding and Training

Nurse leader onboarding and training about sensitive topics is beneficial for setting nurse leaders up for success (Baggett et al. 2016; Li and Gephart 2023; Gabele et al. 2023). A study of novice nurse leaders at a North Texas nonprofit healthcare system who completed a nurse leadership workshop demonstrated increased confidence and competence after workshop completion (Baggett et al. 2016). Results from this small study found that an organized workshop was beneficial for novice nurse leaders (Baggett et al. 2016).

A quantitative cross‐sectional study of nurse leaders was completed from the 2018 National Sample of Registered Nurses in the United States to determine factors for nurse leader turnover (Li and Gephart 2023). Leaders with high levels of dissatisfaction and the inability to operate to the extent of their knowledge, education, and training were more likely to express turnover intentions (Li and Gephart 2023). Providing training for new nurse leaders may help with retention.

Finally, a study of California nurse leaders illuminated a knowledge gap surrounding substance use disorder (SUD) and how to support those nurses as they complete treatment programs and rehabilitate (Gabele et al. 2023). Tailored educational efforts focused on supporting bedside nurses who exhibit signs of SUD are needed (Gabele et al. 2023). Given that substance use disorder is associated with nurse suicide, the results of this study demonstrate an opportunity for nursing leader training on this sensitive topic (Barnes et al. 2022; Gabele et al. 2023).

#### 1.4.1 Somatic Exercises

When educating nurse leaders about sensitive topics, it is important to integrate a process that allows them to refocus their attention on work responsibilities. Somatic exercises can be used to assist this transition. Somatic psychology emphasizes the connection between the mind and body with a distinct focus on how physical sensations and bodily experiences impact the regulation of the nervous system (Levine 2012; Ogden and Fisher 2015). By incorporating body‐centered practices, somatic psychology interventions like movement, tapping, and breathwork aim to downregulate the sympathetic nervous system and engage the parasympathetic nervous system (van der Kolk 2014). Teaching individuals how to perform these interventions fosters repair and recovery after an acute or chronic traumatic event(s) like discussing suicidality.

This paper's purpose is to report the results of a project conducted to enhance nurse leader self‐efficacy and knowledge around suicide prevention strategies to support suicidal healthcare team members.

### 1.5 Design

An evidence‐based practice (EBP) project for nurse leaders was implemented to build skills in supporting team members with suicidal thoughts or behaviors. An interactive 90‐min virtual synchronous training program was created at a large academic hospital system in Southern California. The goal of this project was to increase nursing leaders' self‐efficacy and knowledge surrounding suicide prevention for team members. Nurse leaders were defined as assistant nurse managers, nurse managers, nursing directors, and the chief nursing officer.

The PICO question for the project was: Among nurse leaders at an academic healthcare system in California, does the provision of an educational program using role‐playing practice and creation of a suicide prevention toolkit versus no standard education or training improve self‐efficacy and knowledge on how to take action with a team member who is suspected of being suicidal or voicing suicidal ideation? The definition of “take action” was defined as staying with the team member, calling the suicide crisis hotline, and removing access to means if the team member has a plan.

## 2 Methods

The framework for this EBP project was developed using the Revised San Diego 8 A's Evidence‐Based Practice Model (Ecoff et al. 2020). The 8 A's is an EBP process that provides an eight‐step process for evidence‐based projects, including the catalyst (the problem being addressed by the 8 A's), assessing, asking, acquiring, appraising, applying, analyzing, advancing, and adopting (Ecoff et al. 2020). The San Diego 8 A's model is used throughout the region where this project was completed to guide evidence‐based change and is adopted as the official change model by the project site.

Joanne Duffy's Quality Caring Model was the framework for this EBP project (Duffy 2022). Caring relationships are at the core of the model (Duffy 2022) and were central to how the training program was established. Nursing leaders were encouraged to approach their at‐risk team members with care (Duffy 2022). Nurses work in a community. They develop relationships while at work. Leaders who build caring relationships with colleagues can help nurses feel valued and supported. Nurses, like all people, need to feel cared for to care for themselves. Once nurses feel cared for in the workplace, they are more likely to embrace healthy behaviors that aid in healing, which Duffy describes as a self‐advancing system (Duffy 2022). The hope was that skill‐building with leaders would provide them with a greater capacity to engage in the moment with an at‐risk team member.

The curriculum for the suicide prevention program included risk factors for suicide, at‐risk behaviors, empathic listening techniques, motivational interviewing goals, and available resources. The program also provided an opportunity for role‐playing a conversation with an at‐risk individual, debriefing about their experiences, and finishing with a somatic exercise. The nursing leaders were given prompts to practice during the role play, which included the words an employee might use to indicate risk. The leaders practiced responses to these prompts in small groups. The presentation and script were provided to participants and stored on a shared leadership website of resources after program completion.

The materials included in the nurse leader suicide prevention program were adapted from the ANA informational website for nurse suicide prevention and resilience and suicidality videos from the Ohio State University website with permission from the video creator Sharon Tucker, DNP, RN (American Nurses Association 2023; The Ohio State University 2024; S. Tucker, personal communication, January 15, 2024).

The nurse leaders completed three surveys to assess suicide prevention self‐efficacy and knowledge. The first survey was completed before the suicide prevention training. The second survey was completed immediately after the nurse leaders completed the training. The final survey was completed 1 month after the training.

### 2.1 Recruitment

Participants were recruited through email, word of mouth, meeting presentations, and encouragement from several nursing directors at the academic health system.

### 2.2 Ethical Considerations

This EBP project received Academic Medical Center approval with deferral from the University Institutional Review Board. Data were securely stored in the University‐approved, password‐protected electronic survey platform, and responses to the surveys were anonymous.

### 2.3 Project Sample

The sample included 60 participants employed at an 800+ bed academic healthcare system in Southern California. Forty‐six participants met the inclusion criteria and were identified as assistant nurse managers, nurse managers or nurse supervisors, nursing directors, or chief nursing officers.

### 2.4 Measures

#### 2.4.1 Suicide Prevention Self‐Efficacy

The general self‐efficacy scale (GSE) was used to determine nursing leader's self‐efficacy with suicide prevention (Schwarzer and Jerusalem 1995). The GSE has 10 questions based on a Likert scale with a scale range of 10–40 (Schwarzer and Jerusalem 1995). The GSE has demonstrated internal reliability with Cronbach's alphas between 0.76 and 0.90 (Schwarzer and Jerusalem 1995).

#### 2.4.2 Suicide Prevention Knowledge Assessment

No validated tool to assess the knowledge of nursing leaders of suicide prevention for team members was found in the literature. Eight open‐ended knowledge questions were developed by the first author. The questions were validated using the context validity index (CVI) (Lynn 1986). The questions and a list of acceptable answers were sent to six suicide prevention experts for review. Each question was scored on a scale from 1 to 4, with 1 = not relevant and 4 = highly relevant to the project aim. For the knowledge assessment questions and total questionnaire to be valid, five of the six raters needed to agree that the item was relevant to the aims of the project, and the CVI for each question and the total questionnaire needed to be greater than 0.78 (Lynn 1986). Experts were also asked if the answers were clearly worded with only one meaning and if the list of possible answers was complete. Possible answers for six questions were revised with expert feedback. Two rounds of validation were performed to yield a total score for the knowledge assessment questionnaire of 0.97875 (Table 1).

**TABLE 1:** Validated knowledge assessment questions.

Questions	Correct answers
What are some risk factors for suicide in nurses?	Exposure to repeated trauma Scheduling long, consecutive shifts Repeated requests for overtime Workplace violence, incivility, and bullying Inadequate self‐care Isolation from family and friends Fearing for one's safety or the safety of loved ones Financial stressors Access to and knowledge of lethal substances Constant, high workplace stress Loneliness after relocation, transfer, or new job Issues with management Work/life role conflict Feeling unsupported in the role Feeling like you don't belong Feeling unprepared for the role Fear of harming a patient Being evaluated for substance use disorder Depression Discipline by either Board of Nursing or employer Leaving the job or profession early due to discipline, chronic pain, illness, injury Unmanaged grief in either work or home life Fearing for one's safety or the safety of loved ones Anxiety Night shift Work setting safety/unsafe work environment Misusing substances and afraid will be found out
2 What are examples of at‐risk behaviors?	Talking, writing, or creating art about wanting to die or to kill themselves Looking for a way to kill themselves; searching online for a method/plan Talking about feeling hopeless or having no reason to live Talking about feeling trapped or in unbearable pain Talking about being a burden to others Increasing the use of alcohol or drugs Acting anxious or agitated Sleeping too little or too much Rumination: Cannot get bad thoughts out of their head Withdrawing or isolating themselves Showing rage or talking about seeking revenge Giving away belongings Extreme mood swings Reckless behavior Self‐injurious behavior such as cutting It feels like this person needs more help than a friend can provide Sudden change in mood
3 Case Study: You have a team member who has had issues with absenteeism and you are conducting your time and attendance conversation with the team member. The team member shares with you that their father has cancer and that they are the primary care giver for their father. How do you respond to this team member?	Empathic listening (acknowledge, without judgment; don't try to fix it; connect, be present and vulnerable; bear witness) Educate about EAP or FMLA as an option An “I hear” statement reflecting back on what you heard them say. “I hear you are having a rough time providing care for your father” An empathic statement, such as “I've never been through that myself but it must be so challenging to work while caring for your father with such a serious illness” Be direct—“I know things seem very dark or hopeless to you now yet I know this can turn around and I am here to listen and help”
4 Please answer the following 2 questions based on the following scenario: Team member: Sorry, I had to call out this weekend. I just had a miscarriage a couple of days ago. Manager: That's too bad. At least you know you can get pregnant. What is WRONG with this communication?	Manager uses “at least” statement with team member Manager minimizes team member's experience Manager's own discomfort and lack of empathic skills
5 Please answer the following 2 questions based on the following scenario: Team member: Sorry, I had to call out this weekend. I just had a miscarriage a couple of days ago. Manager: That's too bad. At least you know you can get pregnant. How might you respond differently?	Use empathic listening I'm sorry to hear that. That must have been hard for you. I don't even know what to say. I'm just glad you told me. How can I support you best? I am here to support you however I can.
6 Define in your own terms the definition of “motivational interviewing” and why it might be helpful when engaging with an employee exhibiting behaviors suggestive of suicidal intent.	Collaborative, goal‐oriented communication technique with a focus on change Strengthen personal motivation for and commitment to specific goal Core skills of motivational interviewing: open questions, affirmation, reflections, summarizing Uses an individual's internal motivations to change Know an individual has it within themselves the ability to change Encourages an individual to go away from suicidal thoughts and change toward wanting to get help
7 What should you do when you are with a team member that you feel might be at‐risk for suicide?	Stay with team member, empathic listening, connecting team member with safety plan (who/what comforts you during difficult times, have you contacted them, do you see a therapist, do they know how you feel) Connecting to care/treatment if team member validates actual suicide intent (walk to emergency department if inpatient, call crisis line together)
8 Write in below the actual phone numbers for 3 resources to contact to connect a team member to care.	HEAR program Rachael: 858–657‐6799 or Courtney: 858–657‐6795, hear@health.ucsd.edu or hear.ucsd.edu Employee Assistance Program (EAP): (866) 808–6205 Emergency department/house supervisor could be an answer for inpatient managers National Crisis Hotline: 988 or text “Home” 741–741 SD Crisis Hotline: 1–888–724‐7240

*Note*: Question 1 and 2—Responses including any # and variation of correct answers listed would count as a correct answer; Question 3—Responses including empathic techniques would count as a correct answer; Questions 4 and 5—Responses including a variation of correct answers listed would count as a correct answer; Question 6—Responses including a correct description of motivational interviewing and how it would help a suicidal individual would count as a correct answer; Question 7—Responses including actions to take with a suicidal team member would count as a correct answer; Question 8—Responses including the actual contact information for at least three resources would count as a correct answer.

## 3 Results

### 3.1 General Self‐Efficacy

**FIGURE 1:**
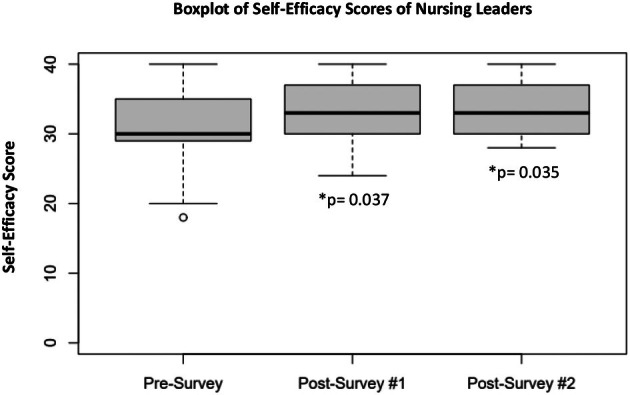
Nursing leaders' increase in self‐efficacy scores. Average scores: Presurvey: 31.3 (*n* = 46), Postsurvey #1: 33.49 (*n* = 36), Postsurvey #2: 33.77; Postsurvey #1 = Immediately following intervention; Postsurvey #2 = 1‐month postintervention; Scale range = 10–40. Range of scores decreases over time, with mean moving in the right direction demonstrating retention of confidence.

There was a statistically significant increase in self‐efficacy scores for the three surveys (Figure 1). The mean self‐efficacy score for the presurvey was 31.3 (*n* = 46, min: 18, max: 40). The mean self‐efficacy score for postsurvey #1 was 33.49 (*n* = 37, min:24, max:40). The mean self‐efficacy score for postsurvey #2 was 33.77 (*n* = 31, min: 28, max:40). As the data for the three surveys were not paired and the requirements of parametric analysis were not met, nonparametric methods were used to determine statistical significance. Self‐efficacy was statistically different following the intervention (*X* ^2^ = 8.0184, *p* = 0.01815).

A post hoc analysis using Dunn's Kruskal–Wallis multiple comparisons with Benjamini–Hochberg adjustment demonstrated that baseline self‐efficacy was significantly lower than both postassessments (adjusted *p* of 0.03711 for presurvey vs. postsurvey #1, and 0.03581 for presurvey vs. postsurvey #2). No significant difference was observed between the two postintervention self‐efficacy surveys (*p* = 0.71675).

### 3.2 Knowledge

**FIGURE 2:**
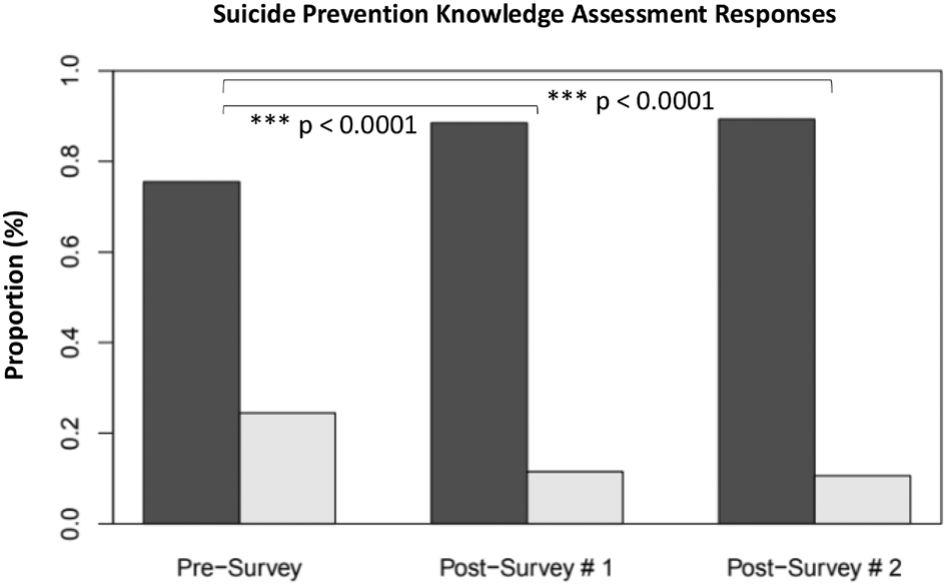
Proportion of incorrect answers on each survey. Dark Bar = Percent correct answers; Light Bar = Percent incorrect answers; Postsurvey #1 = Immediately following the intervention; Postsurvey #2 = 1‐month postintervention.

Suicide prevention knowledge of the nursing leaders was evaluated through the proportion of incorrect answers submitted for the knowledge assessment questions. The proportion of incorrect answers was 24.5% for the presurvey (*n* = 46), 11.5% for postsurvey #1 (*n* = 37), and 10.7% for postsurvey #2 (*n* = 31), respectively (Figure 2). It has been observed that the proportion of incorrect answers varied significantly among the three surveys (*p* = 0.000001). The pairwise comparison reveals that there is a significant difference between the presurvey and postsurvey #1 (*p* = 0.0034) and the presurvey and postsurvey #2 (*p* = 0.00704). However, no significant difference was found between postsurvey #1 and postsurvey #2 (*p* = 0.923). The proportion of incorrect answers is statistically significant at the postintervention (*p* = 0.000001, *X* ^*2*^ = 23.195, df = 2).

### 3.3 Participant Feedback

Though not asked, participants disclosed anecdotal feedback about having had a conversation with a suicidal individual prior to participating in this suicide prevention program. Participants were also given an opportunity to submit feedback after the training. There was overall positive feedback, with common themes of practical information, enjoyment of small groups, and engaging role play. Examples of feedback are included in Table 2. All (*n* = 55, 100%) participants recommended the program to others.

**TABLE 2:** Participant feedback.

Participant feedback
“Great job with the resources provided!”
“I enjoyed the small group format and interactive discussions.”
“Amazing space and program to talk through challenges we have all faced as managers. Great to understand that we are also not alone if a staff member comes to us asking for help.”
“As we discussed in the meeting, healthcare workers are at higher risk for SI and mental health problems. These are really important tools that healthcare staff in a leadership role should be familiar with because you never know when someone is going to confront you with their feelings. Knowing what to do helps us help them. Thank you so much!”
“Great practical information. Particularly liked the role play.”
“This was an excellent training session! I hope down the line, this can be available for all nurses.”
“Hmm. It excellent. The issue of ‘leader vs. staff boundaries’. i.e. confidentiality, ‘I'm not your therapist, but I can help you get to yours or one’.”
“Absolutely wonderful topic! Appreciate the role playing aspect as well. Would like to hear more about the topic we touched on toward the end of class on how to be empathetic, but not take on other's emotions.”
“Have training for all staff. Liked the videos and role play situation.”
“Thank you for sharing this training. Definitely a much needed course for suicide prevention.”
“I thought this would be more triggering having lost a family member to suicide, but it wasn't. You did an amazing job.”
“Thank you for helping to address this key topic and helping to give leaders the resources they need to feel better prepared.”
“This is just a needed training! Great job ***. Maybe we can expand to team leaders like our charge nurses.”
“This was a wonderful exercise—I appreciate the role playing to practice all the skills learned.”
“Sample scenarios were helpful to illustrate how to handle crisis situation. The breakout session also provided opportunities to explore unexpected situations such as refusal of help/assistance.”
“Thank you! It was easy to digest and had great takeaway skills.”

### 3.4 Leader Exposure to Conversations About Suicide

Given the anecdotal feedback gained during the training program, though unplanned a priori, authors used the opportunity at a national conference through audience polling to ask nurse leaders whether they had experience speaking with a colleague about suicide. There were 143 audience members who responded to the polling questions at the American Organization for Nursing Leadership (AONL) conference in April 2024. Of the 143 participants, 58% (*n* = 83) responded that they had direct experience with discussing suicidal ideation or self‐harming behaviors at work, and 13% (*n* = 19) responded that they had not, but upon reflection, they probably should have had the conversation. When asked about having had the conversation at home, 77% (*n* = 110) of the nursing leaders responded that they had had the conversation, and 6% (*n* = 9) responded that they had not, but that they probably should have.

## 4 Discussion

### 4.1 Self‐Efficacy Significance

The nursing leader's self‐efficacy related to suicide prevention was high for all three surveys. This might be attributed to The Joint Commission's (2024) National Patient Safety Goal #15 which requires suicide screening for high‐risk patients. The organization where this program was implemented exceeds these requirements by applying suicide screening to all patients. The nursing leaders at this medical center were familiar with suicide prevention screening for patients through previous training. During the program debriefs, some nursing leaders provided anecdotal feedback that they had experienced conversations with a suicidal individual at work and/or in their personal lives, which may have also elevated the baseline score.

The statistically significant increase in the nursing leader's suicide prevention self‐efficacy suggests that the program was effective. These findings are echoed in previous literature highlighting the importance of suicide prevention trainings for different specialties (Magness et al. 2023; Muehlenkamp and Quinn‐Lee 2023; Stover et al. 2023; Yonemoto et al. 2019; Sylvara and Mandracchia 2019).

The second postsurvey was completed 1 month after the training program. An opportunity for future programs to assess retained self‐efficacy later, after providing enough time to practice skills in the workplace, may yield additional increases in self‐efficacy like those seen in the study of university students (Muehlenkamp and Quinn‐Lee 2023).

### 4.2 Knowledge Significance

#### 4.2.1 Risk Factors and At‐Risk Behaviors

There was already a high level of knowledge of risk factors and at‐risk behaviors. Like suicide prevention self‐efficacy, nursing leaders were familiar with risk factors and at‐risk behaviors from assessing suicide risk in patients (The Joint Commission 2024).

Despite the high baseline, there was an increase in knowledge after program completion. Leaders received additional education about risk factors and at‐risk behaviors during the suicide prevention program. They had access to the teaching materials after program completion for reference.

#### 4.2.2 Empathic Communication

Scores related to empathic communication knowledge were high pre and postintervention. A potential rationale is that empathy is regularly used with patients and team members. Empathy is taught as part of health care professional curriculums (Peisachovich et al. 2023; Samarasekera et al. 2023). In addition, the strong inculcation of Joanne Duffy's Quality Caring Model (Duffy 2022) within the organization may have enhanced leaders' knowledge of empathetic communication.

#### 4.2.3 Motivational Interviewing

The nursing leaders scored the lowest on the motivational interviewing question at all time points. However, knowledge of motivational interviewing increased in the postsurveys. The literature suggests that motivational interviewing results in increased suicide prevention self‐efficacy (Czyz et al. 2018) and increased mental health follow‐up for individuals with suicidal ideation (Lundahl et al. 2024). The low scores on this question demonstrate an opportunity to enhance education on motivational interviewing and provide additional examples in the curriculum to expand nurse leader's knowledge of how to apply the technique in practice.

#### 4.2.4 How to Have a Conversation

While some leaders expressed familiarity with having a conversation with a suicidal individual prior to attending this program, there was an increase in knowledge about how to have a conversation with an at‐risk team member after program completion. Attendees benefited from using a guided script and practicing the conversation with each other in a safe and controlled environment during virtual breakout sessions. Active and live learning are effective tools for increasing suicide prevention awareness (Stover et al. 2023). These results align with current evidence that suggests suicide prevention training leads to better preparedness in intervening with an at‐risk individual (AHA 2023; Magness et al. 2023; Muehlenkamp and Quinn‐Lee 2023; Stover et al. 2023; Sylvara and Mandracchia 2019; Yonemoto et al. 2019).

#### 4.2.5 Suicide Prevention Resources

There was an increase in the correct identification of three resources after program completion. The leaders were given an opportunity to enter the resources' phone numbers into their phones during training, for future easy access. Leaders could reference the phone numbers during the surveys, as they were already on their phones. Being able to successfully retrieve these numbers during the posttest suggests that they complied with this request and were able to find them when needed (Table 1). This was an important step in the program, as easy recall of resources during a conversation with an at‐risk team member will allow for one less thing to think about during a highly stressful interaction.

### 4.3 Feedback

A common theme throughout the training sessions was the anecdotal feedback of participants who had experienced having a conversation with a suicidal individual in both the work setting and their personal lives. Even though leaders mentioned having the conversation prior to this program, 100% (*n* = 55) of program participants said they would recommend the suicide prevention program to others. Despite the high initial and postsurvey suicide prevention GSE scores, there was a statistically significant increase in suicide prevention GSE and knowledge of suicide prevention resources and interventions for an at‐risk team member after program completion. Participant feedback on the postsurvey was positive, and some leaders suggested the program be available for more nurses. These results demonstrate that the nursing leaders felt the program was meaningful and that they benefited from their time spent completing the activities (Table 2).

Results from polling the audience at the 2024 AONL conference confirm the anecdotal feedback about leaders having experience conversing with suicidal individuals. These findings justify the time spent in training managers to prepare for the likely event of having a conversation with an at‐risk individual. Future research is indicated in this under‐studied area of practice.

### 4.4 Somatic Exercise

Nurse leaders participated in a somatic exercise at the end of the suicide prevention training to close the session. The somatic exercise was not mentioned in the participant feedback on the postsurvey because the survey was completed before the exercise. However, some participants verbally expressed appreciation for the reset prior to returning to their daily responsibilities. Finishing with a somatic exercise may have helped the nursing leaders downregulate their sympathetic nervous system and engage their parasympathetic nervous system to support recovery after the sensitive discussions (van der Kolk 2014). There is an opportunity for future studies to measure whether a somatic exercise is beneficial.

### 4.5 Quality Caring Model

The data from this project demonstrate that this program supported nursing leaders to gain self‐efficacy and knowledge to help a team member in need. Theoretically, offering the curriculum is an act of caring for leaders, and deploying the skill with an employee should make the employee feel cared for, with the goal that they would be more likely to take action and seek help. This program created a process where the nursing leaders developed skills to enhance caring relationships for their team members. This project helped nursing leaders build skills to help a suicidal team member feel cared for so that they would seek help, which Duffy (2022) refers to as a self‐advancing system. In previous studies, team members stated that they felt cared for at work when managers saw them as complete humans and inquired about their problems both at home and in the workplace (Baggett et al. 2016).

### 4.6 Interpretation

The AHA (2023) recommends suicide prevention training to be incorporated in healthcare settings. The ANA and the American Academy of Nursing also provide recommendations on suicide prevention (American Nurses Association 2023; Schimmels et al. 2023). Suicide prevention training programs for community members, pharmacists, university students, and faculty have been shown to be successful in suicide prevention preparation (Magness et al. 2023; Muehlenkamp and Quinn‐Lee 2023; Stover et al. 2023; Sylvara and Mandracchia 2019; Yonemoto et al. 2019). This is the first known suicide prevention training targeted at nursing and nursing leaders. The positive recommendation of the program for others, the statistically significant increases in suicide prevention self‐efficacy and knowledge, and the positive feedback the participants shared about their experience with the program support the need to continue to provide education about suicide prevention for nurse leaders.

### 4.7 Limitations

Several limitations to this project should be mentioned. The project was completed at a single site with a small sample size of 46 nursing leaders and 60 total participants. Participation for the 1‐month postsurvey also decreased. Because the surveys were anonymous and nonpaired, parametric statistics could not be used for analysis. Future projects would benefit from multi‐site implementation, larger sample sizes, pairing of the anonymous responses, and a longer follow‐up for the second postsurvey.

### 4.8 Conclusion

A program to train nurse leaders how to engage with team members exhibiting suicidal behaviors is a feasible action leaders can take in response to the call to action from the Surgeon General, Academy of Nurses, and National Academy of Medicine and Science to address suicide prevention in the workplace (The Surgeon General 2021; Schimmels et al. 2023; National Academy of Medicine 2024).

Nursing leaders feel better prepared to support at‐risk team members after completing the suicide prevention skill‐building training program. The synchronous learning method and role‐playing exercises were integral in creating a meaningful experience for the nursing leaders. The results of this project demonstrate a need to include suicide prevention and mental health support training as a required educational competency for nurse leaders. In addition, this education should be added to nurse leader onboarding. Increased nurse leader knowledge and self‐efficacy can help leaders feel better prepared to support team members who are at risk of suicide. A suicide prevention program like this one can easily be modified for nurses at all levels from prelicensure through senior leadership.

## Conflicts of Interest

The authors declare no conflicts of interest.

## Sigma Theta Tau International Chapter

Kristina E. James—Mu Omega, Judy E. Davidson—Gamma Gamma, Julia Rogers—Mu Omega, Patti Ludwig‐Beymer—Mu Omega.

## Clinical Resources

This American Foundation of Suicide Prevention (AFSP) website contains valuable information and videos related to healthcare professionals' mental health and suicide risk: https://afsp.org/healthcare‐professionals‐mental‐health‐and‐suicide‐risk/. The videos posted publicly on this site, hosted by The Ohio State University, were used during the training curriculum: https://u.osu.edu/cliniciansindistress/videos/.

**Nursing Continuing Professional Development**

Journal of Nursing Scholarship is pleased to offer readers the opportunity to earn nursing continuing professional development (NCPD) contact hours for select articles. Sigma Theta Tau International Honor Society of Nursing (Sigma) is accredited as a provider of nursing continuing professional development by the American Nurses Credentialing Center's Commission on Accreditation.

## Data Availability

The data that support the findings of this study are available from the corresponding author upon reasonable request.
